# Evaluation of product conceptual design based on Pythagorean fuzzy set under big data environment

**DOI:** 10.1038/s41598-022-26873-w

**Published:** 2022-12-27

**Authors:** Lian-Dan Ma, Wei-Xing Wang, Jing-Wen Xie, Ning Zhang, Ning-Feng Hu, Zi-Ao Wang

**Affiliations:** 1 College of Mechanical Engineering, Guizhou University, Jiaxiu South Road, Guiyang, 550025 Guizhou China; 2 Key Laboratory of Advanced Manufacturing Technology of Ministry of Education, Guizhou University, Jiaxiu South Road, Guiyang, 550025 Guizhou China

**Keywords:** Electrical and electronic engineering, Mechanical engineering

## Abstract

The concept design evaluation phase of the new product launch is extremely important. However, current evaluation information relies mainly on the a priori knowledge of decision makers and is subjective and ambiguous. For this reason, a conceptual design solution decision model based on Pythagorean fuzzy sets in a big data environment is proposed. Firstly, we use the ability of big data to mine and analyze information to construct a new standard for product concept design evaluation in the big data environment. Secondly, the Pythagorean fuzzy set (PFS), Analytic Hierarchy Process (AHP), and Technique for Order Preference by Similarity to Ideal Solution (TOPSIS) are integrated into a decision model. AHP, extended by the Pythagorean fuzzy set, is used to determine the weights of new conceptual design criteria in a big data environment. The Pythagorean fuzzy TOPSIS is used to prioritize alternative conceptual design solutions. The feasibility of the approach is proven with a practical case, the generalizability of the method is confirmed with two descriptive digital cases, and the reliability, validity, and superiority of the process are demonstrated with sensitivity analysis, comparative analysis, and computational complexity analysis.

## Introduction

The full life cycle of a product can be divided into seven stages: “concept, detail, development, debugging, release, iteration and obsolescence”^1^. As such, product development begins with the design of the concept. Specifically, concept generation and evaluation are two key steps in the product design phase to achieve the best possible design outcome, with the former generating a conceptual design with possibilities and the latter determining the final choice of design candidates^2^. It is well known that successful concept evaluation leads to perhaps disruptive innovation and huge success, whereas poor conceptual evaluation can not only increase design costs and development cycles but also cause additional revisions, and iterations and even jeopardize overall product development success^3^. Given its impact on all succeeding stages in the process of product development, concept evaluation is considered to be one of the most significant activities in product design^4^.

Evaluation of product concept designs is a complex procedure that requires consideration of technological developments, design constraints, user satisfaction and other factors. One of the commonest methods of concept design evaluation is Multi-Criteria Decision Making (MCDM). In traditional conceptual design evaluation methods, the results depend on the subjective judgement of the decision maker, and the designer’s judgement of the conceptual design is subject to uncertainty and lag due to slow research feedback. The majority of current cases show that the evaluation criteria and performance assessment of concept designs rely more on the personal judgement and qualitative descriptions of experienced experts. However, these judgements and descriptions are often subjective, imprecise and sometimes inconsistent due to individual cultural backgrounds, life experiences, logical thinking and other factors. Unreliable decision data early in the design process will lead to almost irreparable design flaws^5^. Extensive research on decision making for conceptual design has found that Jing and others^6^ and others have summarised MCDM methods for conceptual solutions into three types, one is to build pairwise comparison matrices to obtain the weights of evaluation criteria by calculation, for example, BWM (Best Worst Method)^7^ and AHP (Analytic Hierarchy Process)^8^ can deal with the extent to which different assessment criteria influence each other, but are susceptible to the subjective preferences of decision makers. The alternate approach is to combine the assessment figures across various criteria to generate a summed assessment value for each assessment option, and to calculate the combined indicator values to derive the option ranking results, like VIKOR (Vlsekriterijumska Optimizacija I Kompromisno Resenje)^9,10^ and TOPSIS (Technique for Order Preference by Similarity to Ideal Solution)^11^, this type of method does not capture the impact of each evaluation criterion on the overall design. The third type is characterised by an order of preference, which identifies the strengths and weaknesses of different solutions to arrive at the best solution, such as ELECTRE (Elimination Et Choix Traduisant La Realité)^12^ and PROMETHEE (Preference Ranking Organization Method for Enrichment Evaluation)^13,14^, but they cannot deal directly with uncertainties and have limitations in solving realistic decision problems. To address these issues, Zadeh^15^ proposed a fuzzy set theory to deal with imprecise or vague information. And Yager^16^ proposed Pythagorean fuzzy sets (PFS) which are an extensible version of fuzzy sets and intuitionistic fuzzy sets, which can give experts much more liberty to represent judgments on uncertainty and vagueness of decision problems. Akram et al.^17^ combined HYBRID TOPSIS and ELECTRE I solutions with Pythagorean fuzzy information to investigate failure modes and risk factors in impact analysis, PFS extends the linguistic variable hierarchy of IFS (Intuitionistic Fuzzy Sets) to increase the fuzzy information and the acceptable space of data, Pythagorean fuzzy hybrid Order of Preference by Similarity to an Ideal Solution (PFH-TOPSIS) was proved to be a source of highly effective and simple way.

AHP and TOPSIS, due to their ease of computation, unlimited and flexible compatibility with other techniques, and their strong capabilities in analyzing complex decisions and dealing with multiple decision makers, the integrated AHP-TOPSIS approach can take full benefit of these advantages when faced with multiple evaluation criteria and contradicting parameters in product concept design evaluation. The classical AHP-TOPSIS is suitable for numerically precise scenarios, however, in real evaluation environments, many cases cannot be outlined with precise values. In the assessment process, the semantic concept is often a gradual process rather than an abrupt change. For example, if a rating of 5 is meant to be “superior”, then a rating of 5 is “excellent” and a rating of 4.9 is “not superior”, but in reality, in the user’s understanding, a rating of 5 is not very different from a rating of 4.9. In practice, there is no big gap between 5 and 4.9 in users’ understanding^18^. Pythagorean fuzzy sets can be used to depict linguistic variables and express fuzziness, and integrating Pythagorean fuzzy sets into AHP-TOPSIS can enhance the objectivity of product concept design evaluation results.

Technically, assessment criteria can be summarized based on expert experience, literature review^4^, and questionnaires^18^, however, the use of generic data will further increase the uncertainty and imprecision of assessment results. With the further development of social media, wearable devices, and smart manufacturing, massive quantities of databases are coming from all directions. The potential use of vast sums of data is quickly making big data and big data analytics a powerful tool for research teams to develop new applications in new fields. For example, the introduction of big data into healthcare has made a huge leap from traditional to digital healthcare. Researchers have used big data to develop large real medical data platforms^19^, build medical data analysis models for intelligent identification and diagnosis of diseases^20^, and also design medical big data ecosystems on Hadoop big data platforms to provide individualized patient health control and also facilitate the management of patients by medical staff^21^. The combination of data analytics and mobile cloud computing has spawned new research in the field of transportation. Studies have shown that fuzzy Markov prediction models can also be used to forecast efficient short-term traffic^22–24^, offering better route mapping and mobility of cargo and people for better informed and more efficient decisions for transport regulation or development and maintenance of transport structures. Another interesting application area is sentiment analysis, where applying big data to online learning users for sentiment analysis can optimize the learning experience^25^. The way decision makers make decisions are constantly changing and now relies heavily upon creating In today’s competitive environment, companies are not only interested in the technology of big data analytics, but increasingly in how they can use the data they have to create potential value and use this information effectively in their strategies, operational decisions, and innovation processes.

In summary, to attenuate the influence of subjectivity and ambiguity in product concept design evaluation and to achieve comprehensiveness and accuracy in product concept design evaluation, we propose a systematic Pythagorean fuzzy set-based group decision making method by combining Pythagorean fuzzy set-based AHP and TOPSIS in an e-business big data environment, which combines big data, Pythagorean fuzzy sets, AHP and TOPSIS. First, we use a web crawler to crawl big data of users’ online reviews, and then analyze the online reviews using a mean clustering algorithm (K-means) to establish new evaluation criteria. Then, Pythagorean fuzzy sets are fused with hierarchical analysis to calculate the weights of each evaluation criterion; finally, Pythagorean fuzzy sets are combined with ideal solutions to calculate and rank the evaluation results. Based on this method, more accurate and objective data can be obtained for the evaluation of product concept design. The method proposed in this study can also provide a quantitative reference for manufacturers and designers to screen out product design solutions with high user satisfaction.

The motivations for the study in this paper are:

1. We incorporate a thorough and efficient Pythagorean fuzzy set-based approach for product concept design evaluation under a big data environment.
2. In the proposed approach, the evaluation messages of decision makers are provided by Pythagorean fuzzy linguistic variables.
3. The suggested approach incorporates the superiority of big data in treating messages, the superiority of Pythagorean fuzzy sets in dealing with issues of uncertainty, the superiority of AHP among multiple criteria, and the superiority of TOPSIS in decision problems.
4. In the process of product concept design evaluation, PF-AHP-TOPSIS quantifies qualitative information, reduces the serious gap between objective assessment and subjective environment, and makes the PF-AHP-TOPSIS model more logical and useful.

The achievements of the present study are as listed below:

1. Providing a highly efficient, rational, and functional decision-making method for group multi-criteria decision making in a big data environment. Based on big data technology, user preferences and usage habits can be captured in real-time and precisely, thus driving product concept design evaluation.
2. Integrating PFAHP and PFTOPSIS (PF-AHP-TOPSIS) methods as decision models to attenuate the subjectivity and fuzziness of decision makers in the decision-making process. A wealth of expansion of the TOPSIS method in theory and practice.
3. The comparative analysis with PFAHP-FTOPSIS model and PFAHP-PFVIKOR model proves the usefulness and superiority that the raised decision model and the sensitivity analysis is executed by altering the binary weights of the evaluation criteria to ensure the stability of the proposed decision model. Through simulation experiments, it is justified that the proposed model has low computational complexity, and the applicability of the proposed method is further illustrated with the assistance of two numerical cases in addition to the example study.

The rest of this paper is structured and presented below: “Literature review” section, the introduction of proposed product concept design evaluation method in “Proposed design concept evaluation method” section, a practical case study and two illustrative numerical cases for the proposed method are in “An empirical case study” section, sensitivity analysis, comparative analysis, computational complexity analysis, advantages and discussion are in “Analysis and discussion” section, and conclusions and clarification of recommendations for future research in “Conclusion” section.

## Literature review

### Concept design evaluation methodology

Conceptual design evaluation can determine the final choice of alternatives and is the classical MCDM decision problem. In recent years, investigators presented diverse solutions to the concept design evaluation issue. Nghiem and Chu^26^ proposed to combine AHP with ELECTRE I method to solve the problem of evaluating and weighting various criteria and sub-criteria. Wang and Hsueh^27^ proposed a hybrid framework combining AHP, the Kano model, and DEMATEL (Decision Making Trial and Evaluation Laboratory) for incorporating client preference and sensing into product configuration. which incorporates customer preference and perception into product configuration) for discovering ideas for next-generation products. Worsdorfer^28^ developed an analytical model based on AHP that prior to evaluation quantifies the fitness of innovative production concepts at a given scale. The developed model was used to select more promising production alternatives, providing both a fuller and faster procedure for deciding on investments. Prabhat et al.^29^ assigned quantitative weights to user requirements (customer requirements) and product feature quality level (feature quality level) by using AHP assessment, assigning structured weights as opposed to the haphazard values given to designers, and then the structural weights given are applied to both PROMETHEE, which selects the best concept for product development considering both the user and manufacturer perspectives. Hayat et al.^30^ developed a combination of soft set, TOPSIS, and Shannon entropy in order to derive the optimal concept at a range of requirement tiers a promising framework is developed based on soft sets, TOPSIS, and the Shannon entropy. Quan et al.^31^ proposed the KE-GRA-TOPSIS method, which integrates KE (Kansei Engineering), AHP, entropy, game theory, and GRA-TOPSIS (Grey Relation Analysis—TOPSIS) five methods. It can help customers to select the most suitable product according to their subjective needs. Arbelaez et al.^32^ used crowdsourcing augmented reality environment for the evaluation of the esthetics of the product at the concept stage. Liu et al.^33^, in a scientific survey, reviewed breakthrough innovation research, integrated concept evaluation methods from related fields, and developed a breakthrough evaluation method to be employed for product evaluation at the concept design stage.

However, the evaluation data for the conceptual design decision process is mainly determined by the subjective judgment of the decision maker, and precise values can hardly adequately reflect the fuzzy and subjective nature of the decision process. In order to attenuate the influence of these uncertainties on conceptual design evaluation, fuzzy sets have been introduced into conceptual design decision models. Table 1 shows how fuzzy sets and their combined methods have been studied in the field of product design in recent years.

**Table 1:** A brief summary of research on fuzzy sets in the field of product design.

Study	Version of fuzzy set	Combined method	Specific applications
Liang, XD^34^	Fuzzy set	BWM, VIKOR	Evaluate bike-sharing service
Liu, AJ^35^	Fuzzy set	DEMATEL, KMA and VIKOR	Green product collaboration design
Wang, TX^2^	Fuzzy set	Natural Language Processing, TOPSIS	Product evaluation
Mistarihi, MZ^36^	Fuzzy set	ANP, QFD	Modified manual wheelchair design
Jing, LT^37^	Intuitionistic Fuzzy Set	Rough set	Conceptual design evaluation
Li, M^38^	Intuitionistic Fuzzy Set	Kano model, AHP, and QFD	New product development
Feng, CH^39^	Intuitionistic Fuzzy Set	–	Sustainability in product
Hayat, K^40^	Intuitionistic Fuzzy Set	Bijective soft set	Design concept evaluation
Buyukozkan, G^41^	Intuitionistic Fuzzy Set	TOPSIS	Product development
Li, YP^42^	Intuitionistic Fuzzy Set	Karnik–Mendel algorithms, TOPSIS	Modularisation design
Chen, RY^43^	Intuitionistic Fuzzy Set	Tree induction	Product design
Aguirre, PAG^44^	Pythagorean Fuzzy Set	FMEA, DA	Product design

Although the methods of concept design evaluation have been continuously optimized, the evaluation criteria used in these studies are still mainly based on traditional survey methods such as expert opinion^34,36,44^, literature review^4,34,37,41^ and questionnaires^18,35^, which are feasible but have obvious drawbacks such as time-consuming, slow feedback, low user involvement, and small research These methods are feasible but have obvious drawbacks, such as time-consuming, slow feedback, low user participation, and small scope. A prerequisite for effective methods to obtain accurate and objective product concept evaluation results is the establishment of comprehensive and objective evaluation criteria. Without accurate assessment criteria as a basis for evaluation, the scientific validity of product concept design evaluation will be compromised. Big data provides new opportunities and research conditions for product design, and research methods that explore entirely new areas from small-scale data are being gradually replaced by big data parsing^45^. Studies have shown that online review data can be used as a source of information that represents a wide range of user perspectives and is more reliable than user data obtained from other sources, and that product manufacturers can also use online reviews to make quick and favorable decisions and gain a competitive edge in the marketplace^46–48^. Compared to the biases in traditional methods, web-based text mining can directly, quickly, and extensively collect user opinions and obtain a meaningful and complete vocabulary, and the vocabulary collected and the large amount of data involved can compensate for the biases in traditional methods. These words can directly and effectively reflect information about the user’s preferences for the product, which in turn facilitates the evaluation process.

Looking at the above studies, we find fewer studies applying Pythagorean fuzzy sets to product concept design evaluation, both from a fuzzy set methodology perspective (recent studies combining fuzzy sets used as shown in Table 1) and from an application perspective. Given the superior performance of Pythagorean fuzzy sets in dealing with uncertainty problems, the superiority of AHP in dealing with hierarchical relationships of evaluation criteria, the advantages of TOPSIS in decision problems, and the outstanding performance of Big Data in acquiring information and information analysis, this paper proposes a systematic, Pythagorean fuzzy set-based MCDM method in a Big Data environment to fill the gaps in existing research.

### Pythagorean fuzzy set

Pythagorean fuzzy sets are extensibility of fuzzy sets and intuitionistic fuzzy sets, breaking the limitation that the total of the affiliation and insubordination degrees of intuitionistic fuzzy sets must be equal to 1, dealing with uncertainty more reliably and reducing imprecision and ambiguity in the decision making in the course^16^. In a Pythagorean fuzzy set, the sum of the squares of the affiliation and non-affiliation degrees is less than or equal to 1, which is defined as follows:

#### Definition 1

^49^: Let set *X* be a given universe of discourse, and *P* be a Pythagorean fuzzy set (PFS) on the universe of discourse:

1 $$P=\left\{\langle x,P\left(u\left(x\right),v\left(x\right)\right)\rangle |x\in X\right\},$$

where, *u*(*x*) and *v*(*x*) respectively represent the membership degree and non-membership degree of *x*ϵ*P* in the universe *X*, and satisfy $\forall$ *x*ϵ*X, u*(*x*) and *v*(*x*)ϵ[0,1], then:

2 $$0\le {u\left(x\right)}^{2}+{v\left(x\right)}^{2}\le 1.$$

For $\forall$ *x*ϵ*X,* the calculation formula of hesitation degree is:

3 $$\pi \left(x\right)=\sqrt{1-{u\left(x\right)}^{2}-{v\left(x\right)}^{2}}.$$

#### Definition 2

^49^: Let *p* = *P*(*u,v*) be any Pythagorean fuzzy number (PFN), then:

4 $${p}^{c}=P\left(v,u\right).$$

#### Definition 3

^50^: Let *α* = (*u*_*α*_,*v*_*α*_) be PFN, then Eq. (6) is defined as the score function of

5 $$s\left(\alpha \right)={u}_{\alpha }^{2}-{v}_{\alpha }^{2}.$$

#### Definition 4

^35^: Let *α*_*i*_ = (*u*_*αi*_,*v*_*αi*_)(*i* = 1,2) be PFN:

(i) If *s*(*α*_1_) < *s*(*α*_2_) then *α*_1_ *≺α*_2_.
(ii) If *s*(*α*_1_) *≈* *s*(*α*_2_) then *α*_1_∽ *α*_2_*.*

#### Definition 5

^35^:Let *α* = (*u*_*α*_,*v*_*α*_)*, α*_*i*_ = (*u*_*αi*_,*v*_*αi*_)(*i* = 1,2) be PFN, then:

6 $${\alpha }_{1}+{\alpha }_{2}=\left(\sqrt{{u}_{\alpha 1}^{2}+{u}_{\alpha 2}^{2}-{u}_{\alpha 1}^{2}{u}_{\alpha 2}^{2}},{v}_{\alpha 1}{v}_{\alpha 2}\right),$$

7 $${\alpha }_{1}\times {\alpha }_{2}=\left({u}_{\alpha 1}{u}_{\alpha 2},\sqrt{{v}_{\alpha 1}^{2}+{v}_{\alpha 2}^{2}-{v}_{\alpha 1}^{2}{v}_{\alpha 2}^{2}}\right),$$

8 $$\lambda \alpha =\left({\sqrt{1-\left(1-{u}_{\alpha }^{2}\right)}}^{\lambda },{v}_{\alpha }^{\lambda }\right),$$

9 $${\alpha }^{\lambda }=\left({u}_{\alpha }^{\lambda },{\sqrt{1-\left(1-{v}_{\alpha }^{2}\right)}}^{\lambda }\right).$$

### Industrial big data

In manufacturing, Big Data refers to a large amount of multi-source, heterogeneous data generated throughout the product lifecycle^51^. Since its introduction, the concept of big data has been widely used in decision-making^52^. It is often used in engineering research for urban planning^53–56^, energy management^57–60^, smart manufacturing^61–64^, and product development^65–67^. Big data can be classified into the following five categories according to data sources^68^: (i) management data collected from manufacturing information systems; (ii) user data collected from social networking platforms and e-commerce platforms; (iii) device data collected from smart factories; (iv) product data collected from smart products and product service system terminals; and (v) public data collected from governments and agencies. Raw data is multi-scale and highly noisy in addition to being multi-source and heterogeneous and must be processed to obtain the implied information. Partitioned clustering methods divide data objects into clusters of a single structure, and the K-means algorithm is one of the most classical partitioned clustering algorithms.

Under a big data environment, a huge amount of data can improve decision making ability and deliver well data support for decision making, while the real application generates data with unknown, blurred, and missing values due to the unpredictability of the environment, uneven environmental parameters, unstructured database architecture, and other unnecessary reasons. Pythagorean fuzzy sets help to minimize the redundancy and inconsistency of data information and reduce the hazard and decision making of big data information due to their eminent ability to handle uncertain information, missing information, and quantitative data. We discuss multi-criteria decision making in the big data environment and propose a numerical decision model based on Pythagorean fuzzy sets, which improves the accuracy of multi-criteria decision making in the big data environment.

## Proposed design concept evaluation method

Concept design evaluation is designed to guide the design of a product by picking the most potential solution from among the concept solutions. In order to acquire objective and accurate evaluation outcomes, a new framework for product concept design evaluation is provided in this paper. The framework consists of two phases: in the first phase, text mining techniques are used to capture review data from user review big data and process the data information, TFIDF (Term Frequency-Inverse Document Frequency) algorithm is used to calculate text vocabulary weights, K-means algorithm is used to classify review text information, and the classified review text is sorted by designers to establish an evaluation criteria system. The details of the first phase are described in “Text data mining and clustering” section. The evaluation criteria obtained based on big data avoid the uncertainty and imprecision brought by the generic evaluation criteria and lay a solid foundation for obtaining objective evaluation results, and the selection of evaluation criteria from users’ own words is more helpful for users to understand the semantics of the evaluation criteria.

**Figure 1:**
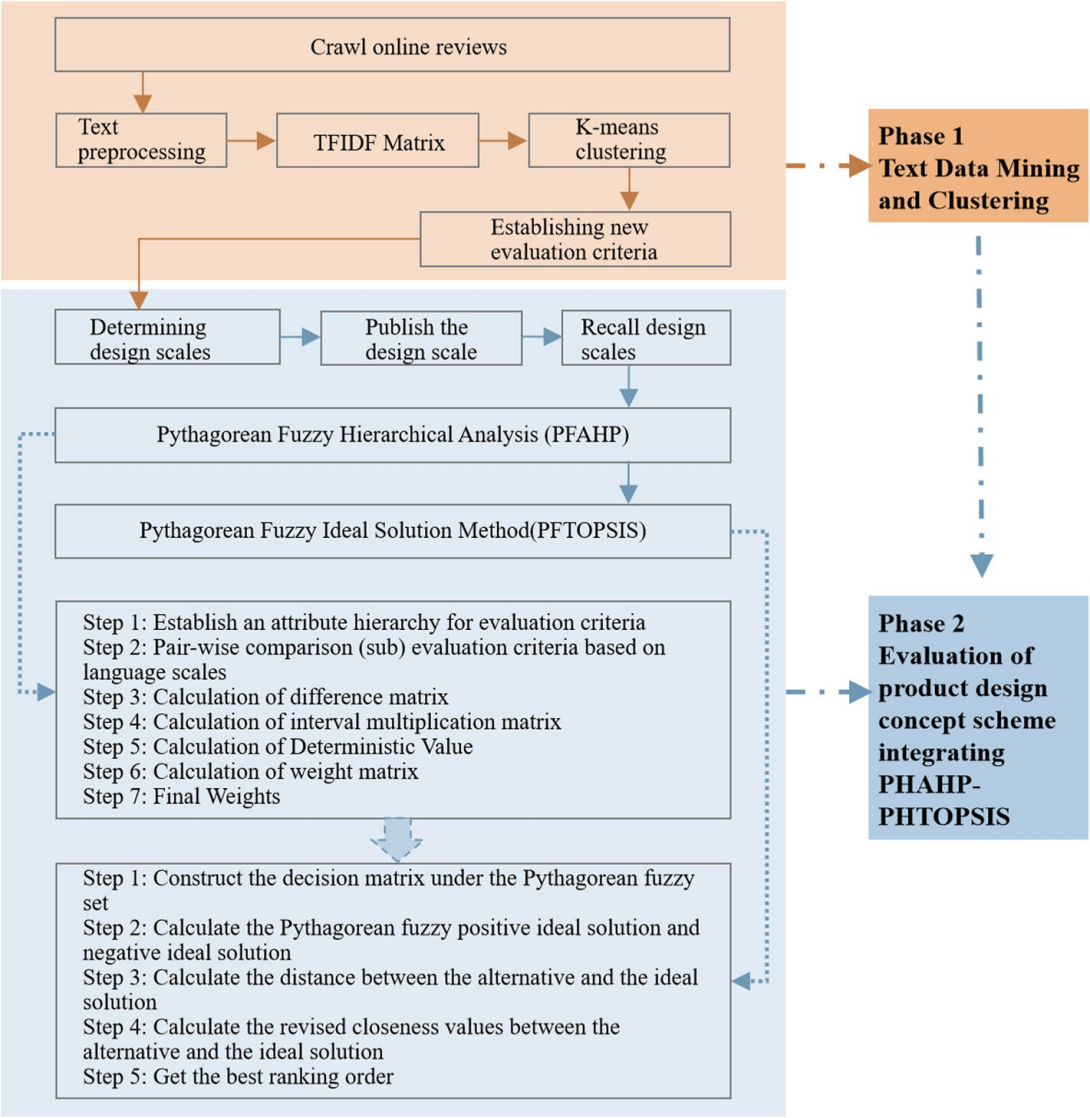
Product concept design assessment framework diagram.

The second stage is to construct numerical models to make decisions on assessment information, and this paper integrates Pythagorean Fuzzy Hierarchical Analysis (PFAHP) and Pythagorean Fuzzy Ideal Solution (PFTOPSIS) into a new decision model. After the experts judge the assessment criteria, the weight values of the assessment criteria are calculated by PFAHP (see “Product concept evaluation weights combined with PFAHP” section for the calculation steps of PFAHP). The constructed evaluation criteria and the concept design solution are designed as a product concept design evaluation questionnaire and published to collect decision data for the concept design decision. The decision data were calculated using PFTOPSIS (see “Optimal product concept evaluation scheme combining Pythagorean fuzzy ideal solution (PFTOPSIS)” section for the calculation steps of PFTOPSIS), and the weight values calculated by PFAHP were quoted in the calculation to finally arrive at the ranking of alternatives. In decision making, Pythagorean fuzzy arrays are used instead of exact numbers, which makes the evaluation less difficult, and at the same time, fuzzy arrays are more compatible with the real-life evaluation environment. The structure of the proposed product concept design evaluation method is shown in Fig. 1.

### Text data mining and clustering

The effective use of user data to evaluate new concept designs is a more feasible approach than traditional user surveys. The specific steps we take to obtain information on user preferences are: first, use python’s requests library to crawl the user comment corpus data, and then use the Jieba library to segment the corpus data; secondly, introduce the Nlp Chinese stop word data set to purify the corpus, and after purification, there is still some interference information in the corpus. The top-ranked interference information is added to the deactivation dictionary, and only words that can reflect the user’s preference are retained. Term Frequency-Inverse Document Frequency (TFIDF) is a statistical algorithm that can evaluate the importance of a word to the total corpus. We use the TFIDF algorithm to obtain the weight of each vocabulary, save the weight results in the form of a matrix, and apply the k-means clustering algorithm (K-means) to classify the corpus. Finally, we perform a simple analysis of the clustering results.

Due to a large amount of data, this paper introduces the SSE (sum of the squared errors, the sum of squared errors) standard to judge the effect of data clustering, analyze the clustering results whether the data within the class is tight and whether the data between classes are separated. The algorithm is as shown in Eq. (10)^69^.

10 $$\mathrm{SSE}=\sum_{i=1}^{k}\sum_{p\in {C}_{i}}{\left|p-{m}_{i}\right|}^{2}.$$

Among them, *C*_*i*_ represents the ith cluster, *p* represents the sample point in *C*_*i*_, *m*_*i*_ is the centroid of *C*_*i*_ (the mean of all samples in it), and *K* is the number of clusters. SSE represents the sum of squared errors of all samples after clustering and their corresponding cluster centers, indicating the accuracy of the clustering results. The higher the degree of aggregation of each class, the smaller the SSE will be, which means that the samples are divided more finely. When the value of *K* is less than the real category, even if *K* is increasing, its increase will greatly increase the degree of aggregation of each cluster, so the SSE will be greatly reduced; when the value of *K* reaches the real category, increase the value of *K* The resulting degree of aggregation decreases rapidly, so the SSE decreases sharply and then flattens as the value of *K* increases.

### Evaluation of product concept design scheme integrating PFAHP-PFTOPSIS

The Pythagorean Fuzzy Set (PFS) is combined with Hierarchical Analysis (AHP) as Pythagorean Fuzzy Hierarchy (PFAHP) for calculating the weights of design concept evaluation criteria, and Pythagorean Fuzzy Set (PFS) is combined with Ideal Solution (TOPSISP) as Pythagorean Fuzzy Ideal Solution (PFTOPSIS). The PFTOPSIS method determines the best ranking of product design concept evaluation solutions by using the weights obtained from PFAHP.

#### Product concept evaluation weights combined with PFAHP

PFAHP is calculated as follows:

- *Step 1* Experts were invited to evaluate the design concept evaluation criteria, which constituted a pairwise comparison matrix *A* = (*a*_*ik*_)_*m*×*m*_, based on the language evaluation of experts, constructed using the scale proposed by Ilbahar et al. (Table 2)^70^.
- *Step 2* The matrix *A* = (*a*_*ik*_)_*m*×*m*_ gives the difference matrix *D* = (*d*_*ik*_)_*m*×*m*_ by Eqs. (11) and (12).
  11 $${d}_{ikL}={u}_{ikL}^{2}-{v}_{ikU}^{2},$$
  12 $${d}_{ikU}={u}_{ikU}^{2}-{v}_{ikL}^{2}.$$
- *Step 3* The difference matrix *D* = (*d*_*ik*_)_*m*×*m*_ is obtained by Eqs. (13) and (14) as the interval multiplication matrix *S* = (*s*_*ik*_)_*m*×*m*_.
  13 $${s}_{ikL}=\sqrt{{1000}^{dL}},$$
  14 $${s}_{ikU}=\sqrt{{1000}^{dU}}.$$
- *Step 4* Use Eq. (15) to calculate the deterministic value *H*=(*h*_*ik*_)_*m*×*m*_.
  15 $${h}_{ik}=1-\left({u}_{ikU}^{2}-{u}_{ikL}^{2}\right)-\left({v}_{ikU}^{2}-{v}_{ikL}^{2}\right).$$
- *Step 5* The determinacy value *H* = (*h*_*ik*_)_*m*×*m*_ is multiplied with the interval multi-plication matrix *S* = (*s*_*ik*_)_*m*×*m*_ according to Eq. (16) to obtain the weight matrix *T* = (*t*_*ik*_)_*m*×*m*_ before normalization.
  16 $${t}_{ik}=\left(\frac{{S}_{ikL}+{S}_{ikU}}{2}\right){h}_{ik}.$$
- *Step 6* Calculate the weight of each criterion using Eq. (17):
  17 $${w}_{i}=\frac{{\sum_{k=1}^{m}t}_{ik}}{\sum_{i=1}^{m}{\sum_{k=1}^{m}t}_{ik}}.$$

**Table 2:** PFAHP’s language terminology scale.

Linguistic term	Pythagorean fuzzy numbers			
	*u*_*L*_	*u*_*U*_	*v*_*L*_	*v*_*U*_
Certainly low important (CLI)	0	0	0.90	1.00
Very low important (VLI)	0.10	0.20	0.80	0.90
Low important (LI)	0.20	0.35	0.65	0.80
Below average important (BAI)	0.35	0.45	0.55	0.65
Average important (AI)	0.45	0.55	0.45	0.55
Above average important (AAI)	0.55	0.65	0.35	0.45
High important (HI)	0.65	0.80	0.20	0.35
Very high important (VHI)	0.80	0.90	0.10	0.20
Certainly high important (CHI)	0.90	1.00	0	0
Exactly equal (EE)	0.1965	0.1965	0.1965	0.1965

#### Optimal product concept evaluation scheme combining Pythagorean fuzzy ideal solution (PFTOPSIS)

The weight value calculated by the PFAHP method is applied to the PFTOPSIS method, and the specific calculation steps are as follows:

- *Step 1* Construct the decision matrix *R* = (*β*_*i*_(*x*_*j*_))_*n*×*m*_ under Pythagorean fuzzy sets. Let the set of assessment options be *X* = {*x*_1_,*x*_2_*,…,x*_*n*_}*,* (*n* ≥ 2)*,* the set of assessment criteria be *β* = {*β*_1_,*β*_2_,…,*β*_*m*_}*,* the weight of each assessment criterion *w* = {*w*_1_*,w*_2_*,…,w*_*m*_}*,* 0 ≤ *w*_*i*_ ≤ 1, and $\sum_{i=1}^{m}{w}_{i}=1$. The assessment value *β*_*i*_ of option *x*_*j*_ criterion is denoted as *β*_*i*_(*x*_*j*_) = (*u*_*ji*_*,v*_*ji*_). Therefore, the decision matrix is:
  18 $$R={\left({\beta }_{i}\left({x}_{j}\right)\right)}_{n\times m}=\left(\begin{array}{cccc}\left({u}_{11},{v}_{11}\right)& \left({u}_{12},{v}_{12}\right)& \cdots & \left({u}_{1m},{v}_{1m}\right)\\ \left({u}_{21},{v}_{21}\right)& \left({u}_{21},{v}_{21}\right)& \dots & \left({u}_{2m},{v}_{2m}\right)\\ \vdots & \vdots & \vdots & \vdots \\ \left({u}_{n1},{v}_{n1}\right)& \left({u}_{n2},{v}_{n2}\right)& \cdots & \left({u}_{nm},{v}_{nm}\right)\end{array}\right).$$
- *Step 2* The Pythagorean fuzzy positive ideal solution (PIS) and the negative ideal solution (NIS) are determined by Eqs. (19) and (20):
  19 $$\begin{aligned} {\text{x}}^{ + } = & \left\{ {{\upbeta }_{{\text{i}}} ,{\text{max}}_{{\text{j}}} \left[ {{\text{s}}\left( {{\upbeta }_{{\text{i}}} \left( {{\text{x}}_{{\text{j}}} } \right)} \right)} \right]{\text{|i}} = 1,2 \ldots {\text{m}}} \right\} \\ = & \left\{ {\left[ {\beta_{1} ,\left( {u_{1}^{ + } ,v_{1}^{ + } } \right)} \right],\left[ {\beta_{2} ,\left( {u_{2}^{ + } ,v_{2}^{ + } } \right)} \right],...,\left[ {\beta_{m} ,\left( {u_{m}^{ + } ,v_{m}^{ + } } \right)} \right]} \right\}, \\ \end{aligned}$$
  20 $$\begin{aligned} x^{ - } = & \left\{ {\beta_{i} ,{\text{min}}_{j} \left[ {s\left( {\beta_{i} \left( {x_{j} } \right)} \right)} \right]{|}i = 1,2...m} \right\} \\ = & \left\{ {\left[ {\beta_{1} ,\left( {u_{1}^{ - } ,v_{1}^{ - } } \right)} \right],\left[ {\beta_{2} ,\left( {u_{2}^{ - } ,v_{2}^{ - } } \right)} \right], \ldots ,\left[ {\beta_{m} ,\left( {u_{m}^{ - } ,v_{m}^{ - } } \right)} \right]} \right\}. \\ \end{aligned}$$
- *Step 3* Use Eqs. (21) and (22) to determine the distance between each evaluation scheme and the Pythagorean fuzzy PIS/NIS.
  21 $$\begin{aligned} D\left( {x_{j} ,x^{ + } } \right) = & \mathop \sum \limits_{i = 1}^{n} w_{i} d\left( {\beta_{i} \left( {x_{j} } \right),\beta_{i} \left( {x^{ + } } \right)} \right) \\ = & \frac{1}{2}\mathop \sum \limits_{i = 1}^{n} w_{i} \left( {\left| {\left( {u_{ji} } \right)^{2} - \left( {u_{i}^{ + } } \right)^{2} } \right| + \left| {\left( {v_{ji} } \right)^{2} - \left( {v_{i}^{ + } } \right)^{2} } \right| + \left| {\left( {\pi_{ji} } \right)^{2} - \left( {\pi_{i}^{ + } } \right)^{2} } \right|} \right), \\ \end{aligned}$$
  22 $$\begin{aligned} D\left( {x_{j} ,x^{ - } } \right) = & \mathop \sum \limits_{i = 1}^{n} w_{i} d\left( {\beta_{i} \left( {x_{j} } \right),\beta_{i} \left( {x^{ - } } \right)} \right) \\ = & \frac{1}{2}\mathop \sum \limits_{i = 1}^{n} w_{i} \left( {\left| {\left( {u_{ji} } \right)^{2} - \left( {u_{i}^{ - } } \right)^{2} } \right| + \left| {\left( {v_{ji} } \right)^{2} - \left( {v_{i}^{ - } } \right)^{2} } \right| + \left| {\left( {\pi_{ji} } \right)^{2} - \left( {\pi_{i}^{ - } } \right)^{2} } \right|} \right). \\ \end{aligned}$$
- *Step 4* Use Eq. (23) to calculate the revised closeness *ξ*(*x*_*j*_) of the evaluation scheme *x*_*j*_*.*
  23 $$\xi \left({x}_{j}\right)=\frac{D\left({x}_{j},{x}^{-}\right)}{{D}_{max}\left({x}_{j},{x}^{-}\right)}-\frac{D\left({x}_{j},{x}^{+}\right)}{{D}_{min}\left({x}_{j},{x}^{+}\right)}.$$
- *Step 5* Finally, the best ranking of product design concept evaluation solutions was determined, and the solution with the highest correction factor was the best.

## An empirical case study

Rapid advances in drone technology and improvements in size, cost, and intelligence have led to a gradual lowering of the threshold for the use of consumer-grade drones, extending their utility in communications, photography, agriculture, surveillance, and various public services^71^. They are also widely sought after in major e-commerce platforms. Therefore, we choose a consumer-grade aerial photography drone as the product for our case study to validate the practicability of the proposed product design concept evaluation model.

Corresponding to the product concept design evaluation framework (Fig. 1), firstly, we crawled and analyzed the reviews of drone consumers from e-commerce platforms to construct a targeted evaluation criteria system, i.e., the content of “Get data sources” section. Second, experts in the field are invited to make independent judgments on the constructed evaluation criteria, and the relative importance of the evaluation criteria is calculated according to PFAHP, i.e., the content of “Weighted calculation of assessment criteria using PFAHP” section. Finally, the three existing drone conceptual design assessment schemes with the constructed assessment criteria system were prepared as online questionnaires and published on the Internet in anonymous form to collect questionnaire data from drone consumers, and the obtained questionnaire data were calculated using the PFTOPSIS method, i.e., the content of “Prioritization of product design concepts using PFTOPSIS” section. An illustrative numerical example is added to “Explanatory numerical examples” section to further illustrate the practicality of the method used. The example study described in detail in this section provides a clear understanding of how the proposed method works in the big data environment based on Pythagorean fuzzy set quantification for the product concept design evaluation process.

### Get data sources

We collect user text data on consumer-grade aerial drones from JD.COM, one of the largest e-commerce websites in China. First, we use a crawler to crawl JD’s high-selling consumer-grade aerial drone reviews, collecting a total of 6741 web text reviews, and then process the data as described in “Text data mining and clustering” section. “Like”, “good”, “received”, “satisfied”, “Buy” and other words are high-frequency words for reviews (n = 1199; 1122; 835; 823 and 432) but they do not reflect users’ preferences for products and are not meant for the actual evaluation. Therefore, in order to avoid their interference with the final statistical results, we added the above words to the deactivated word list for secondary cleaning of the original data, and the total number of valid comments after secondary cleaning was 5697. We conducted a frequency analysis of online reviews of drones to obtain words that clearly express user preferences, and the results of the frequency analysis are summarised in Table 3. There are 24 words with high frequencies that clearly express user preferences, and they appear in the text a total of 9549 times, and the most frequent words are “texture”, “cheap”, “simple” and “clear”.

**Table 3:** Results of frequency analysis of users’ preferred terms from online reviews.

Vocabulary for expressing preference	Frequency	Percentage of total	Vocabulary for expressing preference	Frequency	Percentage of total
Technology	58	0.62	Comfortable	33	0.35
Textured	1467	15.74	Clear	988	10.60
Cool	67	0.72	Rigorous	71	0.76
Aesthetic	519	5.57	Ingenious	187	2.01
Advanced	128	1.37	Stable	730	7.83
Exquisite	367	3.94	Durable	81	0.87
Stunning	62	0.67	Portable	296	3.18
Novelty	41	0.44	Smart	252	2.70
Simple	1095	11.75	Cheap	1213	13.02
Smooth	71	0.76	Energy-saving	737	7.91
Responsive	786	8.43	Professional	71	0.76
Generous	174	1.82	Fashionable	55	0.58

**Figure 2:**
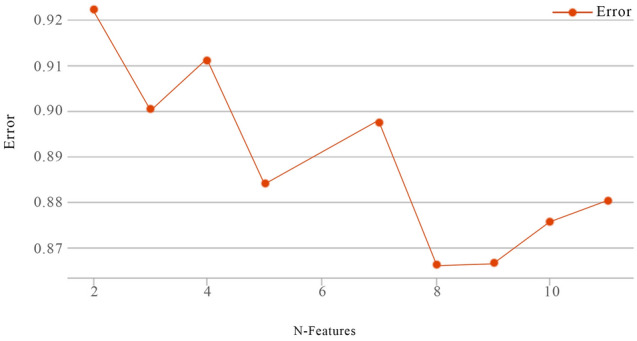
SSE folding line chart.

**Figure 3:**
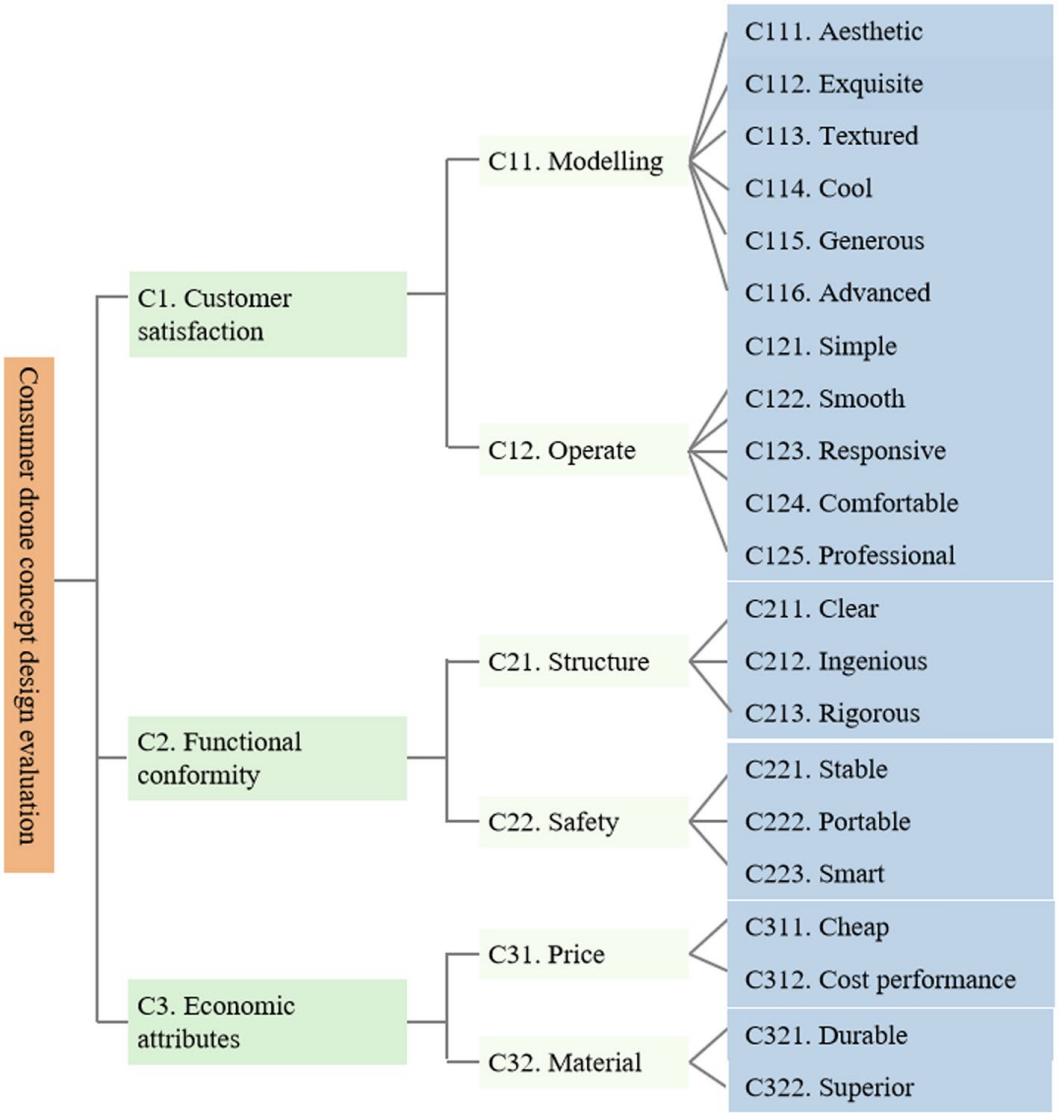
Consumer drone concept design evaluation criteria system.

Taking effective reviews as the source of corpus data, the bag-of-words model selects the top 54 feature words (such as textured, simple, clear, and technology) that have a large TFIDF weight and can centrally reflect user preferences as the k-means clustering basis. The number of k-means user preference optimal clusters is found by the SSE standard between the cluster value of 2 and 11, as shown in Fig. 2. The abscissa of Fig. 2 is the number of clusters, and the ordinate is the average distance of each corpus, and its value can reflect the degree of aggregation of each type. It can be seen from Fig. 2 that when the review samples are divided into 8 categories, the broken line tends to be stable, so we choose 8 as the number of clusters. After determining the number of clusters, we obtained the number of clusters and their central words and sorted the results into Table 4. According to Table 4, we eliminate the comment text in category 1, which has a large amount of data and cluttered categories and merge the comment data in categories 2 and 3, which all point to operability. According to the most representative words of each category and combined with the original corpus, word frequency, and design dimensions, the results are summarized as the design concept evaluation criteria, as shown in Fig. 3.

**Table 4:** K-means clustering results.

Category	Number of comments (n)	Central words (frequency of central words(n))	Representative statements of this category of comments
Cluster01	3815	Textured (328), Stable (316), Simple (276), Clear (239), Smart (167)	Good appearance, excellent texture, the fast logistics and delivery, cheaper than the physical store price
Cluster02	325	Simple (176), Noise (30), Convenient (26)	The drone that I have wanted for a long time is simple to operate, easy to use, easy to carry, the texture is perfect and it has been verified that it is genuine
Cluster03	390	Range (218), Responsive (96), Control (69), Folding (20)	The remote control aircraft has high responsive and long battery life, simple to operate, as described, super fun, great!
Cluster04	213	Simple (133), Stable (121), Novice (37), Lightweight (14)	The shape is very cool, it is quite simple to assemble by yourself, easy to control, and it flies very stably. I like it very much
Cluster05	350	Durable (80), Responsive (72), Smooth (14)	The manufacturing material is very durable, and it is not a problem to occasionally fall from a low place. The flying height and operation are very stable, and the shooting is very high-definition
Cluster06	256	Cost performance (132), Price (111), Textured (94)	Very good, the quality is very good, children love to play, the price is very high, cost performance is well, worth buying
Cluster07	249	Aesthetics (230), Appearance (52), Technology (40), Exquisite (23), Textured (9)	The goods are received, the model is aesthetic and generous, I bought it as a gift for my son, my son said it works great
Cluster08	99	Ingenious (68), Rigorous (38), Price (2)	It is quite simple to operate! The structure is also very Ingenious

### Evaluation of target product design concept solutions

#### Weighted calculation of assessment criteria using PFAHP

Ten experts in the field were invited to pairwise compare the assessment criteria system shown in Fig. 3 using the language terms of PFS (shown in Table 2). The ten experts (including five males and five females with an average age of 35.1) are engineers from different departments with a deep knowledge base in the fields of equipment manufacturing, smart technology, and product design, and they have 9 years (mean) of experience in product development to provide a valid assessment of the evaluation criteria system for this study.

**Figure 4:**
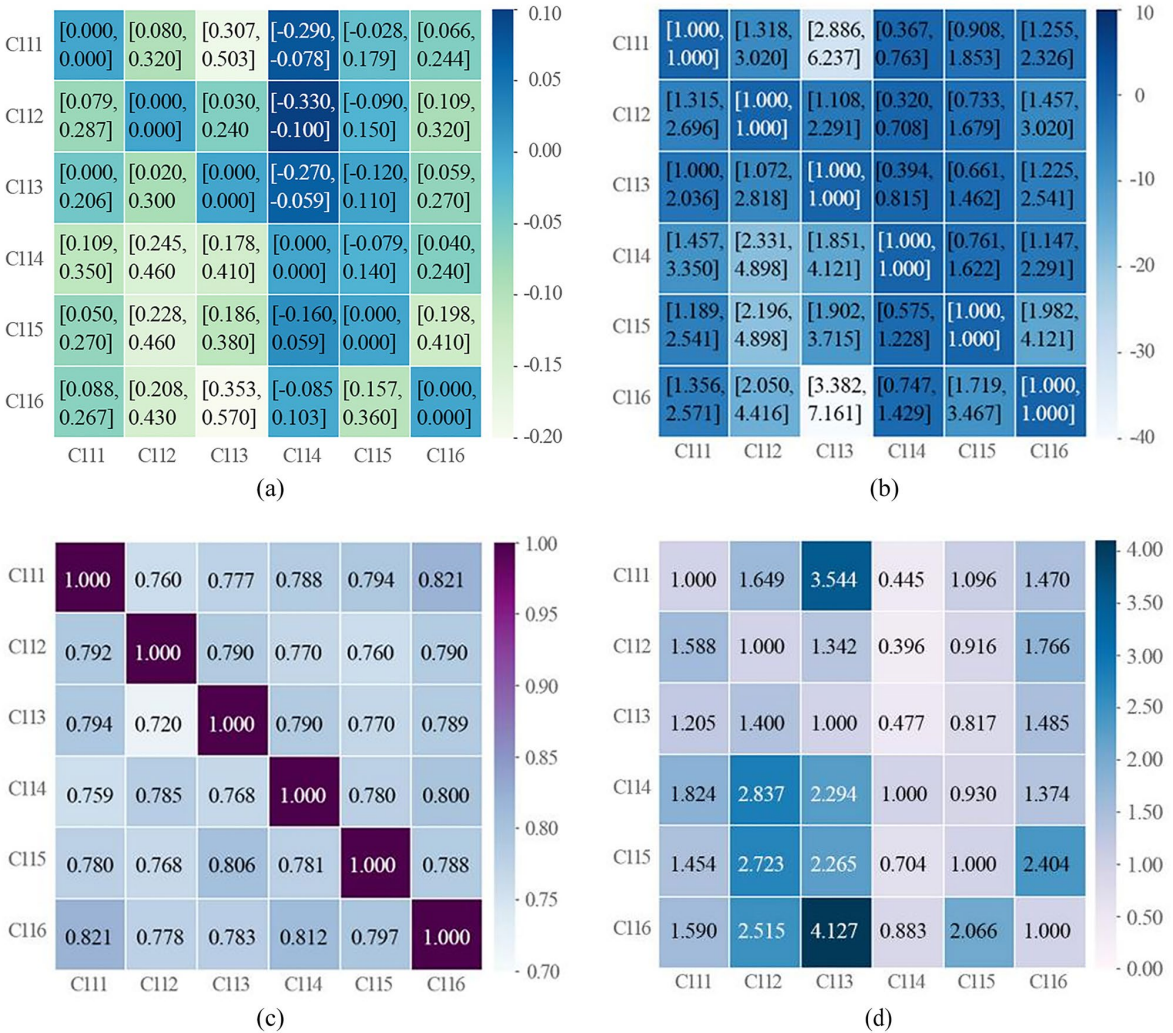
Matrix diagram: (**a**) difference matrix, (**b**) interval multiplication matrix, (**c**) deterministic value matrix, (**d**) weight matrix before normalization.

**Figure 5:**
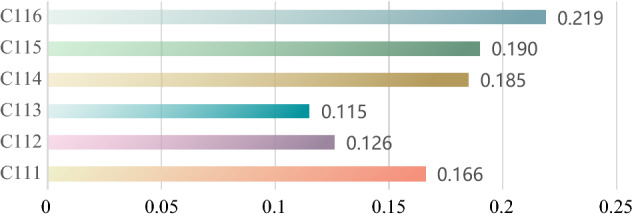
The weights of C111–C116.

In this process, linguistic terms are converted to the corresponding Pythagorean fuzzy interval values. Since these experts make different ratings, their subjective judgments need to be aggregated into a compromise pairwise comparison matrix. In this paper, the most representative data (Tertiary criteria assessment C111–C116) are used as an example to provide the relevant calculation results. Table 5 shows the compromise pairwise comparison matrices of the assessment criteria, and the compromise pairwise matrices of Table 5 are next calculated according to steps 2–6 described in “Product concept evaluation weights combined with PFAHP” section, and the results obtained are the difference matrix (Fig. 4a), the interval multiplication matrix (Fig. 4b), the deterministic value matrix (Fig. 4c), and the pre-normalization weight matrix (Fig. 4d). Figure 5 gives the final weight values calculated by the PHAHP method for C111–C116. The same calculation steps are performed in other evaluation criteria to calculate the local weights and global weights of the evaluation criteria, and the results are listed in Table 6.

**Table 5:** The compromise pairwise comparison matrix of C111–C116.

	C111	C112	C113	C114	C115	C116
C111	{[0.197, 0.197], [0.197, 0.197]}	{[0.540, 0.660], [0.340, 0.460]}	{[0.650, 0.765], [0.235, 0.340]}	{[0.355, 0.450], [0.530, 0.645]}	{[0.450, 0.565], [0.375, 0.480]}	{[0.505, 0.600], [0.340, 0.435]}
C112	{[0.535, 0.640], [0.350, 0.455]}	{[0.197, 0.197], [0.197, 0.197]}	{[0.510, 0.620], [0.380, 0.480]}	{[0.335, 0.450], [0.550, 0.665]}	{[0.455, 0.575], [0.425, 0.545]}	{[0.550, 0.660], [0.340, 0.440]}
C113	{[0.500, 0.595], [0.385, 0.500]}	{[0.510, 0.650], [0.350, 0.490]}	{[0.197, 0.197], [0.197, 0.197]}	{[0.365, 0.465], [0.525, 0.635]}	{[0.440, 0.555], [0.445, 0.560]}	{[0.520, 0.635], [0.365, 0.460]}
C114	{[0.550, 0.675], [0.325, 0.440]}	{[0.615, 0.730], [0.270, 0.365]}	{[0.585, 0.705], [0.295, 0.405]}	{[0.197, 0.197], [0.197, 0.197]}	{[0.455, 0.570], [0.430, 0.535]}	{[0.515, 0.620], [0.380, 0.475]}
C115	{[0.525, 0.635], [0.365, 0.475]}	{[0.610, 0.730], [0.270, 0.380]}	{[0.585, 0.690], [0.310, 0.395]}	{[0.420, 0.525], [0.465, 0.580]}	{[0.197, 0.197], [0.197, 0.197]}	{[0.595, 0.705], [0.295, 0.395]}
C116	{[0.535, 0.630], [0.360, 0.445]}	{[0.600, 0.715], [0.285, 0.390]}	{[0.670, 0.785], [0.215, 0.310]}	{[0.425, 0.525], [0.415, 0.515]}	{[0.570, 0.680], [0.320, 0.410]}	{[0.197,0.197], [0.197,0.197]}

**Table 6:** Local and global weights for consumer drone design concept evaluation criteria.

Primary criteria	Secondary criteria	Tertiary criteria	Local weight	Global weight
C1(0.427)	C11(0.486)	C111	0.166	0.034
		C112	0.126	0.026
		C113	0.115	0.024
		C114	0.185	0.038
		C115	0.190	0.039
		C116	0.219	0.045
	C12(0.514)	C121	0.244	0.054
		C122	0.258	0.057
		C123	0.172	0.038
		C124	0.175	0.038
		C125	0.150	0.033
C2(0.268)	C21(0.609)	C211	0.316	0.055
		C212	0.404	0.070
		C213	0.280	0.047
	C22(0.391)	C221	0.319	0.033
		C222	0.283	0.030
		C223	0.398	0.042
C3(0.305)	C31(0.466)	C311	0.554	0.079
		C312	0.446	0.063
	C32(0.534)	C321	0.586	0.095
		C322	0.414	0.067

Table 6 shows the weight values of each evaluation criterion. The results show that the five most important criteria for evaluating consumer drone design concepts are: durable (C321), cheap (C311), ingenious (C212), superior (C322), and cost performance (C312). The five least important The evaluation criteria are: textured (C113), Exquisite (C112), portable (C222), stable (C221) and professional (C125).

#### Prioritization of product design concepts using PFTOPSIS

**Figure 6:**
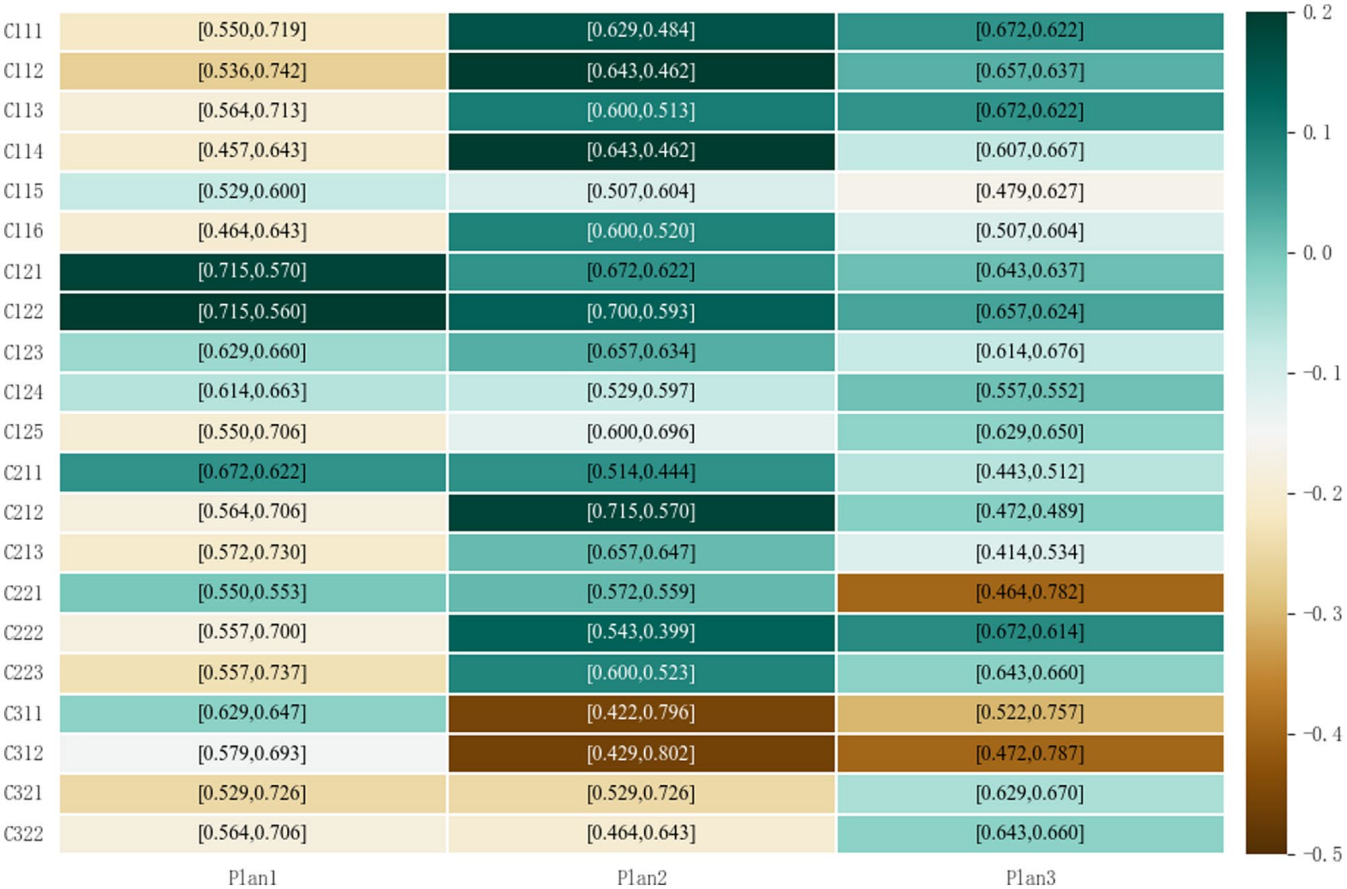
Decision matrix for three design concept plans.

An anonymous online questionnaire was published via the Internet, which was designed according to the language scale of Pérez-Domínguez et al.^72^ (Table 7), and three prone design concept plans were evaluated using a system of evaluation criteria, which are briefly described in Table 8. A total of seven prone consumer responses were collected. The collected response data were collated, the linguistic variables were converted to Pythagorean fuzzy numbers, and then the criteria weights calculated in the PHAHP method were applied to the calculation of the PFTOPSIS analysis. The decision matrix constructed for this evaluation is shown in Fig. 6.

**Table 7:** Pythagorean fuzzy linguistic scale used in PFTOPSIS.

Linguistic term	Corresponding Pythagorean fuzzy number (u,v)
Extremely low (EL)	(0.10, 0.99)
Very little (VL)	(0.10, 0.97)
Little (L)	(0.25, 0.92)
Middle little (ML)	(0.40, 0.87)
Middle (M)	(0.50, 0.80)
Middle high (MH)	(0.60, 0.71)
Big (B)	(0.70, 0.60)
Very tall (VT)	(0.80, 0.44)
Tremendously high (TH)	(0.10, 0.00)

**Table 8:** Basic information on three aerial drone design concept plans.

Number	Name	Design concept description
Plan1	UCO flying camera	Create a unique, smart, portable, long-lasting aerial photography drone. Quickly replace batteries, extend flight time by 45 min, install a mobile app on your phone, control the angle of drone rotation and adjust system settings while seeing what the camera captures
Plan2	Compact folding aerial photography drone	Designed for aerial photography and stabilized video capture, the folding drone can be flown when needed by simply removing it from its hard case and rotating the quadcopter with click action. The drone is equipped with all the advanced sensors needed to navigate in tight spaces or track waypoints via a compatible app on your phone
Plan3	X drone	It is an ultra-portable drone that alleviates the problems of other drones that are difficult to carry, store, assemble and use by matching geometric shapes to produce intuitive graphic elements. The smartphone app provides users with a live view of the onboard cameras and flight data, as well as a simple user interface for controlling the drone

Using Eqs. (19) and (20), the Pythagorean fuzzy PIS and Pythagorean fuzzy NIS values are determined and the obtained results are as follows:

$$x^{ + } = \{ p(0.{629}, \, 0.{484}),p(0.{643}, \, 0.{462}), p(0.{600}, \, 0.{513}),p(0.{643}, \, 0.{462}),p(0.{529}, \, 0.{600}),p(0.{600}, \, 0.{520}),p(0.{715}, \, 0.{570}),p(0.{715}, \, 0.{560}),p(0.{657}, \, 0.{634}),p(0.{557}, \, 0.{552}),p(0.{629}, \, 0.{65}0),p(0.{514}, \, 0.{444}),p(0.{715}, \, 0.{57}0),p(0.{657}, \, 0.{647}),p(0.{572}, \, 0.{559}),p(0.{543}, \, 0.{399}),p(0.{600}, \, 0.{523}),p(0.{629}, \, 0.{647}), p(0.{579}, \, 0.{693}), p(0.{629}, \, 0.{670}),p(0.{643}, \, 0.{660})\} .$$

$$x^{ - } = \{ p(0.{55}0, \, 0.{719}),p(0.{536}, \, 0.{742}), p(0.{564}, \, 0.{713}),p(0.{457}, \, 0.{643}),p(0.{479}, \, 0.{627}),p(0.{464}, \, 0.{643}),p(0.{643}, \, 0.{637}),p(0.{657}, \, 0.{624}),p(0.{614}, \, 0.{676}),p(0.{529}, \, 0.{597}),p(0.{55}0, \, 0.{7}0{6}),p(0.{443}, \, 0.{512}),p(0.{564}, \, 0.{7}0{6}),p(0.{572}, \, 0.{73}0),p(0.{464}, \, 0.{782}),p(0.{557}, \, 0.{7}00),p(0.{557}, \, 0.{737}),p(0.{422}, \, 0.{796}), p(0.{429}, \, 0.{8}0{2}), p(0.{529}, \, 0.{726}),p(0.{464}, \, 0.{643})\} .$$

Using Eqs. (21) and (22), the distances of the alternatives to the Pythagorean fuzzy PIS and NIS are calculated, and the results are provided in Table 9. In addition, the revised closeness values are calculated using Eq. (23), and the results are also shown in Table 9.

**Table 9:** Closeness coefficients of design concept plans.

Evaluation plan	*D*(*x*_*i*_*,x*^+^)	*D*(*x*_*i*_*,x*^*−*^)	$\xi$(*x*_*i*_)	Ranking order
Plan 1	0.130	0.090	− 1.261	2
Plan 2	0.064	0.103	− 0.100	1
Plan 3	0.145	0.115	− 1.272	3

According to the PFTOPSIS method, the evaluated solution with modified discount progress (*x*_*i*_) closest to 1 is the solution closest to the positive ideal solution and far from the negative ideal solution. Therefore, having the largest (*x*_*i*_) value means that the drone solution that is considered by the user to performs best in the conceptual design phase. According to Table 9, Plan 2 is the best conceptual design solution.

### Explanatory numerical examples

#### Case 1: evaluation of the design concept of a garbage container for a kitchen

The characteristics of the kitchen waste container are in some way consistent with the evaluation criteria shown in Fig. 3, such as “the shape is exquisite”, “the structure is clear”, “the material is durable”, etc. We will follow the evaluation criteria shown in Fig. 3 and their weight values (Table 6) to apply the PFTOPSIS model to the conceptual design of kitchen waste containers discussed by Liu et al.^18^. Ten participants were appointed randomly to form a decision panel to express their viewpoints on the conceptual design options in linguistic terms (Table 7) after learning about the four conceptual design options for kitchen waste containers shown by Liu et al.^18^. Table 10 presents the collated decision matrix, Table 11 shows the corresponding Pythagorean fuzzy PIS and NIS, and Table 12 provides the distances of the conceptual design solutions from the Pythagorean fuzzy PIS and NIS, along with the revised closeness of the conceptual design solutions and the final ranking of the solutions.

**Table 10:** Decision matrix.

	Design1	Design2	Design3	Design4
C111	[0.485, 0.415]	[0.643, 0.584]	[0.643, 0.584]	[0.632, 0.522]
C112	[0.530, 0.542]	[0.551, 0.413]	[0.624, 0.567]	[0.532, 0.337]
C113	[0.464, 0.413]	[0.520, 0.513]	[0.523, 0.573]	[0.503, 0.522]
C114	[0.557, 0.563]	[0.567, 0.513]	[0.591, 0.562]	[0.507, 0.567]
C115	[0.539, 0.530]	[0.530, 0.513]	[0.547, 0.564]	[0.540, 0.527]
C116	[0.564, 0.543]	[0.554, 0.533]	[0.601, 0.546]	[0.567, 0.551]
C121	[0.615, 0.589]	[0.574, 0.603]	[0.661, 0.652]	[0.620, 0.637]
C122	[0.723, 0.681]	[0.564, 0.553]	[0.721, 0.693]	[0.657, 0.634]
C123	[0.632, 0.621]	[0.564, 0.533]	[0.637, 0.624]	[0.614, 0.606]
C124	[0.514, 0.503]	[0.524, 0.513]	[0.537, 0.557]	[0.520, 0.552]
C125	[0.559, 0.546]	[0.564, 0.513]	[0.620, 0.650]	[0.612, 0.602]
C211	[0.562, 0.532]	[0.564, 0.543]	[0.574, 0.544]	[0.443, 0.412]
C212	[0.554, 0.526]	[0.554, 0.513]	[0.615, 0.579]	[0.465, 0.465]
C213	[0.589, 0.590]	[0.589, 0.563]	[0.637, 0.597]	[0.414, 0.434]
C221	[0.581, 0.567]	[0.564, 0.513]	[0.586, 0.565]	[0.464, 0.482]
C222	[0.557, 0.560]	[0.564, 0.513]	[0.565, 0.589]	[0.502, 0.514]
C223	[0.557, 0.547]	[0.589, 0.573]	[0.623, 0.599]	[0.601, 0.589]
C311	[0.509, 0.539]	[0.504, 0.513]	[0.522, 0.546]	[0.509, 0.537]
C312	[0.579, 0.593]	[0.564, 0.598]	[0.589, 0.602]	[0.582, 0.601]
C321	[0.529, 0.516]	[0.524, 0.513]	[0.536, 0.526]	[0.529, 0.536]
C322	[0.484, 0.501]	[0.464, 0.473]	[0.489, 0.503]	[0.453, 0.469]

**Table 11:** Pythagorean fuzzy PIS and NIS.

${{x}_{1}^{+}}^{ }=$	$\left\{P\left(0.632, 0.522\right),P\left(0.532, 0.337\right),P\left(0.464, 0.413\right),P\left(0.567, 0.513\right),P\left(0.53, 0.513\right),P\left(0.601, 0.546\right)\right\}$
	$$\left\{P\left(0.615, 0.589\right),P\left(0.723, 0.681\right),P\left(0.564, 0.533\right),P\left(0.524, 0.513\right),P\left(0.564, 0.513\right),P\left(0.574, 0.544\right)\right\}$$
	$$\left\{P\left(0.554, 0.513\right),P\left(0.637, 0.597\right),P\left(0.564, 0.513\right),P\left(0.564, 0.513\right),P\left(0.623, 0.599\right),P\left(0.504, 0.513\right)\right\}$$
	$$\left\{P\left(0.589, 0.602\right),P\left(0.529, 0.516\right),P\left(0.464, 0.473\right)\right\}$$
${{x}_{1}^{-}}^{ }=$	$\left\{P\left(0.485, 0.415\right),P\left(0.53, 0.542\right),P\left(0.523, 0.573\right),P\left(0.507, 0.567\right),P\left(0.547, 0.564\right),P\left(0.567, 0.551\right)\right\}$
	$$\left\{P\left(0.574, 0.603\right),P\left(0.564, 0.553\right),P\left(0.614, 0.606\right),P\left(0.52, 0.552\right),P\left(0.62, 0.65\right),P\left(0.564, 0.543\right)\right\}$$
	$$\left\{P\left(0.465, 0.465\right),P\left(0.414, 0.434\right),P\left(0.464, 0.482\right),P\left(0.565, 0.589\right),P\left(0.557, 0.547\right),P\left(0.509, 0.539\right)\right\}$$
	$$\left\{P\left(0.564, 0.598\right),P\left(0.529, 0.536\right),P\left(0.484, 0.501\right)\right\}.$$

**Table 12:** PFTOPSIS calculation results for the conceptual design solution.

Design	D(*x*_*D*_*,*${x}_{1}^{+}$)	D(*x*_*D*_*,*${x}_{1}^{-}$)	$\upxi$(*x*_*D*_)	Rankig order	Liu et al.^18^ Ranking of managers after assigning confidence levels (managers)
Design 1	0.050	0.082	− 0.361	2	2
Design 2	0.046	0.080	− 0.282	1	1
Design 3	0.072	0.111	− 0.571	3	4
Design 4	0.090	0.065	− 1.369	4	3

The outcomes in Table 12 reveal that Design 2 is the best design and Design 1 is the second best one, which is consistent with Liu et al.’s^18^ ranking of the conceptual design after increasing the confidence level of managers, which indicates the universality of the method proposed in this paper. And there are many potential reasons for the inconsistent ranking of Design 3 and Design 4, for example, changes in the assessment criteria, changes in the relative importance of the assessment criteria, etc.

The method proposed by Liu et al.^18^ requires an extended linguistic scale (from three to five levels) if one wants to consider managers’ influence factors (self-confidence), which undoubtedly increases the subjectivity and ambiguity of the assessment process and increases the probability of distortion of the assessment results, the method proposed in this paper, which uses a uniform linguistic scale for all decision makers, ensures the uniformity of the assessment environment and attenuates the “human influence factors”, and the assessment results are more objective and reasonable.

#### Case 2: conceptual design selection of a smart logistics transport vehicle

At present, traditional logistics vehicles can no longer meet the operational needs of logistics enterprises, so the development of intelligent logistics transport vehicles is very necessary, and the evaluation results have a certain orientation for the development of enterprise products. Therefore, we constructed six evaluation criteria from the perspective of market demand: F1 motor-rated power, F2 wearing parts, F3 aesthetic shape, F4 operation, and maintenance cost, F5 storage capacity, and F6 distribution security. A decision team of 10 people with backgrounds in research and development, manufacturing, and use evaluated the four available options, using linguistic variables to express their views on the evaluation criteria, and the options are chosen.

The weight values *θ* = (0.135, 0.148, 0.132, 0.150, 0.203, 0.231) were calculated by the PFAHP model, and the final results were obtained by the PFTOPSIS model, and the best intelligent logistics transport vehicle concept design option was Option 4, and the specific calculated values are shown in Table 13.

**Table 13:** Ranking of smart logistics transport vehicle concept design options.

Scheme	D(*x*_*F*_*,*${x}_{2}^{+}$)	D(*x*_*F*_*,*${x}_{2}^{-}$)	$\upxi$(*x*_*F*_)	Ranking order
Scheme 1	0.206	0.159	− 1.438	4
Scheme 2	0.170	0.272	− 0.692	3
Scheme 3	0.112	0.227	− 0.287	2
Scheme 4	0.103	0.286	0.0	1

The drone example and two illustrative numerical cases demonstrate the practicality of the approach proposed in this paper.

The decision model generated based on Pythagorean fuzzy sets can be applied in product design not only for conceptual design evaluation, but also for product sustainability selection, product modularity decision, product color evaluation, and other stages of the full product life cycle. In addition to product design, it can be applied to other fields such as material selection, robot selection, and machine tool selection in manufacturing and mechanical engineering, performance and benchmarking evaluation, personnel selection, and business investment decisions in business management, supplier selection and site selection in logistics and supply chain, wastewater management in natural environment and resources, software evaluation, network selection, and website evaluation in information science, website evaluation, etc.

Although Big Data can provide powerful data support for decision making, it cannot avoid the defects of the data itself. Pythagorean fuzzy sets, due to their own characteristics, provide a precise and superior mathematical-logical framework for expressing fuzzy information, which far exceeds the performance of fuzzy sets and intuitionistic fuzzy sets, while also excelling in handling multidimensional data. In short, the integrated method retains the advantages of the approach itself while increasing the scope of its use, and these features prove it to be a reliable method for solving multi-criteria decision problems.

## Analysis and discussion

This section provides further analysis and discussions to illustrate the computational efficiency of the model proposed in this paper, the last subsection presents the advantages of the proposed approach.

### Sensitivity analysis of assessment criteria

In this subsection, a sensitivity analysis of the weights of the assessment criteria is performed to test the stability of the weight calculations. This is followed by an analysis of the impact of the values of the criteria weights calculated by the PFAHP on the ranking of the assessment scenarios according to different scenarios. These scenarios are generated by changing the binary weights of the criteria^73^. Thus, three different scenarios are generated from a combination of three different criteria (C1–C3). The results of the sensitivity analysis calculations are given in Table 14.

**Table 14:** Sensitivity analysis results for different criterion weights.

Scenarios	Criteria weights (wi)			Alternative weights [ξ(xi)]			Ranking of alternatives	Compromise solutions
	C1	C2	C3	Plan1	Plan2	Plan3		
Current	0.427	0.268	0.305	− 1.261	− 0.100	− 1.272	Plan2–Plan1–Plan3	Plan2
C1–C2	0.268	0.427	0.305	− 1.658	− 0.633	− 1.839	Plan2–Plan1–Plan3	Plan2
C1–C3	0.305	0.268	0.427	− 0.633	− 0.262	− 0.676	Plan2–Plan1–Plan3	Plan2
C2–C3	0.427	0.305	0.268	− 1.574	− 0.052	− 1.615	Plan2–Plan1–Plan3	Plan2

The sensitivity analysis shows that even though different weights are assigned to the assessment criteria and different relative postings are obtained, the ranking results are always the same and Plan2 is the best choice in all scenarios, providing strong and reasonable data support to confirm the reliability of the proposed decision model.

### Comparative analysis of decision models

In order to test the validity of the proposed decision model, the results of the model were compared and analyzed with those of the PFAHP-PFVIKOR model and the PFAHP-FTOPSIS model, and the results are shown in Table 15.

**Table 15:** Calculation results of each method.

Evaluation plan	Current method (PFAHP-PFTOPSIS)		PFAHP-PFVIKOR		PFAHP-FTOPSIS	
	$\upxi$(xi)	Ranking order	Q (*v* = 0.5)	Ranking order	$\upxi$(xi)	Ranking order
Plan 1	− 1.261	2	0.154	2	0.2067	3
Plan 2	− 0.100	1	0.037	1	0.2078	1
Plan 3	− 1.272	3	0.173	3	0.2072	2

VIKOR, from the Serbian “VIsekriterijumska optimizacija i KOmpromisno Resenje”, is a decision making method based on ideal points, proposed by Opricovic and Tzeng in 1998. Like TOPSIS, the solution that is closest to the positive ideal solution and furthest from the negative ideal solution is selected as the optimal solution. Following the Pythagorean fuzzy set VIKOR method as extended by Muhammet et al.^74^, we take *v* = 0.5. The ranking order of the best solutions is determined by the minimum value of Q when the two conditions of Awasthia^75^ are satisfied. The first comparative analysis was performed in PFTOPSIS with PFVIKOR and Table 10 shows that it yields a consistent ranking order with the PFVIKOR method, validating the validity of the current method.

The traditional TOPSIS method is only able to be used in numerically accurate situations and the FTOPSIS method is an extension of the TOPSIS method under fuzzy sets. A second comparative analysis was performed between the currently proposed decision model and PFAHP-FTOPSIS^76^. The results show that the ranking order derived using the PFAHP-FTOPSIS model is slightly different from the current integrated approach, with the top ranking still being Plan 2, but Plan 1 and Plan 3 being ranked differently. Some of the reasons for the difference in ranking may be that (i) the subordination of Pythagorean fuzzy sets is more detailed than the subordination of fuzzy sets; (ii) in some cases, intuitionistic fuzzy sets cannot satisfy the condition when the subordination and non-subordination are greater than 1, whereas Pythagorean fuzzy sets can, in the case of Pythagorean fuzzy sets, the sum of squares cannot exceed 1, whereas the sum of subordination and non-subordination can This makes Pythagorean fuzzy sets more sensitive, flexible and powerful in dealing with uncertainty.

The above results demonstrate the validity and reliability of the proposed decision model, which can be used to evaluate product concept designs by taking advantage of the Pythagorean fuzzy set, which has significant advantages over other fuzzy sets in terms of sensitivity in the face of data and in dealing with the uncertainty of the problem, providing more reasonable and accurate results.

### Computational complexity analysis of decision models

In this section, the computational complexity of the proposed decision model is discussed in terms of time complexity and space complexity through simulation experiments. The experimental studies performed are all based on Python 3.7 on an ordinary PC with 12th Gen Intel(R) Core(TM) i7-12700H 2.30 GHz, 16 GB RAM.

As can be seen from Table 16, the computational complexity of PFAHP-PFTOPSIS is simpler than PFAHP-PFVIKOR and Z-AHP-TOPSIS and more complex than PFAHP-FTOPSIS. This is because the Pythagorean fuzzy set divides the linguistic terms more carefully and achieves dimensionality reduction for the data.

**Table 16:** Simulation experiment results data.

	PFAHP-PFTOPSIS	PFAHP-PFVIKOR	PFAHP-FTOPSIS	Z-AHP-TOPSIS
Program running time (S)	0.004988	0.006724	0.004443	0.005013
Program running memory (KB)	116.0	132	95	119

### Advantages of the proposed work

In real-world problems, big data and Pythagorean fuzzy sets are more appropriate design decision tools to address vagueness, subjectivity, and imprecision in concept design evaluation. Pythagorean fuzzy sets provide a reliable mathematical framework in which vague conceptual factors in the product design evaluation process in big data environments can be studied precisely and rigorously. This paper combines AHP, TOPSIS, and PFS to convert qualitative evaluation criteria into quantitative parameters evaluated through product concept design, which is advanced in generating evaluation criteria and evaluating alternatives, showing a distinctive innovation in the design evaluation process, and the advantages of the proposed concept design evaluation method are summarized as follows.

1. The proposed method uses TFIDF and K-means to analyze user review data collected from e-commerce platforms, enabling designers and manufacturers to clarify user preferences and usage habits of products in a comprehensive, real-time, and precise manner, facilitating the analysis and construction of product concept design evaluation criteria that meet user needs and corporate interests, mitigating the impact of cognitive biases of design/manufacturing experts It can also reduce the impact of the cognitive bias of design/manufacturing experts, instead of relying on the experience and intuition of experts.
2. The PF-AHP-TOPSIS decision model uses generalized triangular intuitive fuzzy numbers instead of precise quantitative numbers to express the quantitative assessment of decision makers in the concept design evaluation process, increasing the space for accommodating uncertain information and data, weakening the ambiguity and subjectivity of decision makers, and enhancing the objectivity of evaluation results while avoiding the precise rating of product concept design evaluation criteria. makes the evaluation easier and more flexible.
3. This study provides an effective and practical solution to the complexity of the fuzzy multi-criteria decision-making problem in the industrial big data environment, which is more targeted and more in line with the current decision-making environment, considering both the inherent uncertainty of individual evaluation information and the subjectivity within the decision-making group; the credibility of the ranking results is well enhanced, ultimately providing manufacturers and designers with reasonable and objective evaluation The credibility of the ranking results is well enhanced, ultimately providing manufacturers and designers with reasonable and objective evaluation results.

## Conclusion

Based on fuzzy mathematical theory and the characteristics of online review big data, this study proposes a group decision model applying Pythagorean fuzzy sets for product concept design evaluation, which is investigated in an example with an aerial photography drone. In the flow of the proposed method, user preference data of users for the product are mined and segmented for application to evaluation criteria, and the raw, subjective and uncertain perceptions of decision makers are captured and represented as fuzzy values. In the process of concept design evaluation, individual verbal assessments are converted into fuzzy values, criteria weights are determined through a hierarchical analysis fused with Pythagorean fuzzy sets, and alternatives are ranked through an ideal solution based on Pythagorean fuzzy sets. One of the main features of the proposed approach, which attempts to solve the problem of product concept design evaluation based on the analysis of consumer reviews, is that it does not only attenuate the influence of uncertainties from the perspective of a decision model, but the entire process, the entire product concept design evaluation framework, serves the purpose of achieving more objective and reasonable evaluation results.

Although the proposed method provides a quantitative and reliable objective decision model for the evaluation of product concept design in the example study, there are certain limitations in this study. When the object of application changes, the assessment criteria may follow suit, requiring once again textual information mining, clustering, etc. The second is that there are various e-commerce websites in each country/region, but this study only selected JD.COM reviews, and the evaluation criteria established have limited application. The subjective evaluation of the evaluation criteria and product concept design solutions relies on the experience of experts and users, which may make the evaluation results variable. Further research is, therefore, necessary to capture the expression of user preferences for product attributes in various regions and cultures; it is also possible to weigh up experts or users and set risk parameters to reduce the interference of subjective factors. The PF-AHP-TOPSIS decision model is still somewhat challenging to compute for non-specialists, and subsequent software programs can be developed and promoted to simplify the computation process. When the decision makers’ views tend to be neutral, the evaluation results calculated by the PF-AHP-TOPSIS decision model are difficult to distinguish obviously.

Other methods used to solve the product concept design evaluation problem are VIKOR, ELECTRE, PROMETHEE, BWM, FMEA, etc. These methods have their own characteristics, for example, VIKOR considers the subjective preferences of decision makers, while TOPSIS, which does not consider the subjective preferences of decision makers in the decision making process, has a more powerful performance in excluding humans (experts, consumers, decision makers, etc.) errors and is more in line with the original intention of obtaining objective evaluation results in this paper.

In future research, the construction of evaluation criteria can be further discussed, other methods can be combined with Pythagorean fuzzy sets according to the purpose of decision making to develop more decision models for big data environments, and the proposed methods can be applied to other fields to process multidimensional data so as to obtain reliable decisions. Further extensions of TOPSISI to more complex spherical fuzzy sets can also be investigated, and the advantages and disadvantages of the extensions are discussed to explore their practical applications.

## Data Availability

The datasets generated during and/or analysed during the current study are available from the corresponding author on reasonable request.
